# What Is Adult Hippocampal Neurogenesis Good for?

**DOI:** 10.3389/fnins.2022.852680

**Published:** 2022-04-15

**Authors:** Gerd Kempermann

**Affiliations:** ^1^German Center for Neurodegenerative Diseases (DZNE), Dresden, Germany; ^2^Center for Regenerative Therapies Dresden (CRTD), Technische Universität Dresden, Dresden, Germany

**Keywords:** spatial learning, hippocampus, synaptic plasticity, mouse, human, pattern separation

## Abstract

Adult hippocampal neurogenesis is a unique and exceptional process in the mammalian brain that in a lifelong and activity-dependent way generates new excitatory principal neurons. A comprehensive view on their function in greater contexts has now emerged, revealing to which extent the hippocampus (and hence brain and mind) depend on these neurons. Due to a postmitotic period of heightened synaptic plasticity they bias incoming excitation to the dentate gyrus to non-overlapping subnetworks, resulting in pattern separation and the avoidance of catastrophic interference. Temporally, this promotes the flexible integration of novel information into familiar contexts and contributes to episodic memory, which in humans would be critical for autobiographic memory. Together these local effects represent a unique strategy to solve the plasticity-stability dilemma that all learning neuronal networks are facing. Neurogenesis-dependent plasticity also improves memory consolidation. This relates to the surprising involvement of adult neurogenesis in forgetting, which is also hypothesized to be critically relevant for negative plasticity, for example in post-traumatic stress disorder. In addition, adult-born neurons also directly mediate stress-resilience and take part in affective behaviors. Finally, the activity- and experience-dependent plasticity that is contributed by adult neurogenesis is associated with an individualization of the hippocampal circuitry. While a solid and largely consensual understanding of how new neurons contribute to hippocampal function has been reached, an overarching unifying theory that embeds neurogenesis-dependent functionality and effects on connectomics is still missing. More sophisticated multi-electrode electrophysiology, advanced ethologically relevant behavioral tests, and next-generation computational modeling will let us take the next steps.

## Introduction

**FIGURE 1:**
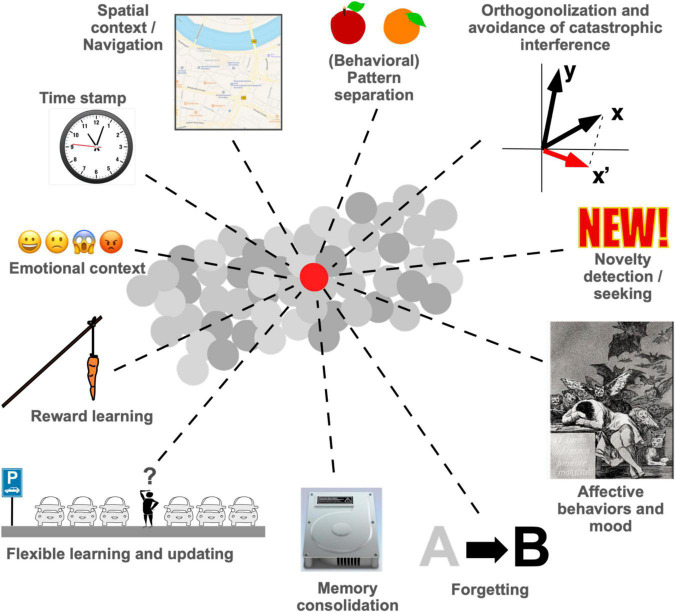
Proposed functions of adult hippocampal neurogenesis in learning and memory as well as in affective behaviors. This list is not exhaustive, but the field is now at a point, at which many of the proposed functions converge. Forgetting and memory consolidation, for example, are clearly related, and so are pattern separation and orthogonalization. We have not yet achieved a unifying theory for all or most of these ideas, but much progress has been made. This article discusses some of the avenues that the field might take to obtain a more integrative view on what new neurons in the hippocampus are good for.

Adult neurogenesis is a fascinating topic that is relevant at a grander scale because the new neurons exert distinct and relevant functions. These functions influence our understanding of cognition and affective behavior and might have important translational consequences. In the past 60 years since the original discovery of adult hippocampal neurogenesis by Altman and Das (1965), a number of different functions that are provided by new neurons have been revealed by increasingly sophisticated experiments. This Perspective article attempts to briefly summarize the state-of-the-art (Figure 1) and to provide an outlook to the remaining challenges. The argument flows from cellular over network to behavioral effects and moves from effects taking place in seconds to a life-course perspective, pointing to some possible ways to integrate concepts across these various domains and scales. As a Perspective article the overview cannot be exhaustive and the path to integration cannot be more than a suggestion, but this article will hopefully nevertheless raise awareness that the apparently divergent ideas about what adult neurogenesis is good for have begun to coalesce into a common framework.

## Where Adult Hippocampal Neurogenesis Takes Place

Adult hippocampal neurogenesis is limited to only one part of the hippocampal formation, the dentate gyrus, which is not even part of the canonical “hippocampus proper.” As the dentate gyrus is a late-evolved mammalian specialty (Treves et al., 2008; Hevner, 2016), adult hippocampal neurogenesis as we see it in mice, the best studied species in this context, is a mammalian specialty as well (Kempermann, 2012). There is, however, adult neurogenesis also in the functionally and structurally homolog regions in birds and fish. Especially the bird studies, for example involving spatial navigation in food-caching birds, suggest that hippocampal function might benefit in more than one way from adding new neurons (Brenowitz and Larson, 2015), but adult neurogenesis in the mammalian dentate gyrus occurs in a much more focused sub-system. The new granule cell neurons selectively contribute to the mossy fiber connection between the dentate gyrus (DG) and area CA3. This structure, especially in its infrapyramidal blade has been known as extremely plastic for a long time (Schwegler et al., 1981; Römer et al., 2011). As adult neurogenesis is regulated by (cognitive) activity, the mossy fiber system and the network in the dentate gyrus are adaptable to an extent not seen in any other context of the adult brain. Within the canonical tri-synaptic backbone of the hippocampus (Entorhinal cortex → DG → CA3 → CA1 Subiculum/Entorhinal cortex) the DG → CA3 sub-system is unique. Excitatory neurons are added to the network—if there is a turnover, it takes place at protracted time-scales. Adult hippocampal neurogenesis is not about replacement but about network plasticity.

## The Local Network Effects of Adult Neurogenesis Support Pattern Separation

Activity in the dentate gyrus is extremely sparse and the network is massively inhibited through a dominance of the local inhibitory interneurons. In their postmitotic development the new neurons go through a phase of increased excitability and enhanced synaptic plasticity (Schmidt-Hieber et al., 2004; Ge et al., 2007). This, together with the close feedback loop they establish with local basket cells, results in biasing the excitation in the network to the new cells and further suppression of the network through lateral inhibition. Through this principle and due to the general sparseness of the overall activity, incoming input (from the entorhinal cortex *via* the performant path) activates neuronal clusters in the dentate gyrus in a non-overlapping way (for review see Lodge and Bischofberger, 2019). This results in the increased spatial separation of input patterns. “Pattern separation” is a cardinal function of the dentate gyrus and research of the past decades has shown that it is achieved through a key contribution of the newborn neurons (Clelland et al., 2009; França et al., 2017). See also Hvoslef-Eide and Oomen for an extensive review on the topic (Hvoslef-Eide and Oomen, 2016).

The insight that new neurons are involved in pattern separation has propelled adult hippocampal neurogenesis from an odd side note to a key feature, underlying a central functionality of the hippocampus (Sahay et al., 2011). Nevertheless, the immediate local network function attributable to increased synaptic plasticity appears to be transient and dependent on a critical window in the course of development of the new neurons (Gu et al., 2012). There are other functional contributions beyond this critical time window (Lemaire et al., 2012), but the lasting network effects are not well understood to date.

In the Morris water maze task, the newborn neurons improved only the hippocampus-dependent aspects of the task performance (Dupret et al., 2008) and improved the flexible use of advanced learning strategies (Garthe et al., 2016). These results link functionality that depends on pattern separation with other aspects of task performance. One of the key challenges for the field is to understand these links and to develop integrative concepts. The proposed functions as in Figure 1 and the additional examples in the text have a common core that the field will have to unravel in the future. Neither are these all separate functions, nor can the be subsumed under pattern separation as a core functionality of the dentate gyrus.

Nevertheless, given the broad acceptance of a role of adult-born neurons in pattern separation, the issue has also particular relevance for adult hippocampal neurogenesis in humans. Adult hippocampal neurogenesis in the human dentate gyrus has repeatedly been questioned because in terms of morphology, marker expression and other aspects, the situation in humans appears to be different from the one in rodents. Because these questions have been extensively discussed elsewhere (Moreno-Jiménez et al., 2021; Sorrells et al., 2021), we will not go into greater detail in this Perspective article on Function. Nevertheless, the fact that, between species, things might look different is a poor argument to support the non-existence of adult neurogenesis in the lesser-studied species. It remains a puzzling question, how the human dentate gyrus would, if those claims were true, achieve pattern separation (and its other functions) without adult neurogenesis.

## Network Complexity Is Still Underappreciated in the Adult Neurogenesis Field

The exact position of the dentate gyrus in the overall hippocampal network is much more complex than the canonical tri-synaptic backbone suggests (Bienkowski et al., 2018). Among them, there are (1) connections between the EC and CA3, bypassing the DG altogether, so that CA3 already “knows” the information reaching CA3 *via* the DG, (2) recurrent connections from CA3 to the DG, and (3) additional pathways between hippocampus and cortex, which might reflect that the DG is, in evolutionary terms, a late add-on. These networks complicate the judgment of how new neurons affect overall hippocampal function. For a review of contributions of adult neurogenesis at the network level, see Tuncdemir et al. (2019).

The dentate gyrus (Kohara et al., 2014) and the newborn neurons (Llorens-Martín et al., 2015) also project to region CA2, which is of particular interest, because CA2 is involved in social memory (Hitti and Siegelbaum, 2014). Social interaction in turn is a stimulus for adult neurogenesis (Moreno-Jiménez et al., 2019) and, finally, an association between adult neurogenesis and social memory has been described (Cope et al., 2020).

## The Temporal Dimension of Pattern Separation

Pattern separation can also be described as the avoidance of catastrophic interference (Wiskott et al., 2006). Many behavioral studies that manipulated adult neurogenesis have shown that adult hippocampal neurogenesis in fact contributes to what is called “behavioral pattern separation” (in contrast to the computational pattern separation which solely looks at the trueness of the outputs compared to the inputs). One aspect of pattern separation is the detection of novelty. Adult hippocampal neurogenesis correlates with novelty seeking in rodents (Lemaire et al., 1999; Denny et al., 2012) and biases attention toward novelty (Weeden et al., 2019). Exposure to learning stimuli and environmental enrichment stimulate adult neurogenesis (Kempermann et al., 1997; Gould et al., 1999; for review see Kempermann, 2015). To identify new things as new is also relevant for the contextualization of information. This has been shown for example in highly reductionistic studies with the paradigm of “contextual fear conditioning,” where new neurons add the memory of the context of the unpleasant stimulus (Saxe et al., 2006; Ko et al., 2009; Gao et al., 2018). In more complex spatial learning tasks, adult neurogenesis allows the integration of new information into previously learned contexts (Garthe et al., 2009; Swan et al., 2014). This reversal learning or updating is an important aspect of cognitive flexibility (Berdugo-Vega et al., 2021). A commonly used example of this is the parking lot analogy: every day you have to remember where exactly your parked your car in the same parking lot. To achieve this, you do not necessarily have to forget where you parked the day before or last Christmas, but you have to distinguish irrelevant past information from relevant new information in a pre-established context that is largely stable (and changing only with respect to features like weather, etc.).

New neurons are a particular way to solve the so-called stability-plasticity dilemma: stable immutable neuronal networks do not forget but cannot learn anything new, while highly plastic networks readily learn new contents but cannot retain them. Computational models revealed that in terms of the lowest error rate and the better efficiency adult neurogenesis can outperform other solutions to this problem (Wiskott et al., 2006).

The resulting separation of bouts of information thus has a temporal dimension. Adult neurogenesis thereby allows ordering information in time (sometimes referred to as applying a “time stamp”), which is a pre-requisite for episodic memory and a cardinal function of the hippocampus (Aimone et al., 2006, 2010). Episodic memory in turn is the foundation of autobiographic memory, which is central to human self-awareness. From that perspective, the question of adult hippocampal neurogenesis in humans seems even more pressing.

## Adult Neurogenesis and Forgetting Versus Memory Consolidation

The link between adult hippocampal neurogenesis and forgetting is particular intriguing and only at first sight counter-intuitive (Akers et al., 2014; Scott et al., 2021). Flexibility over time means that information must also be cleared from the hippocampus not only in order to make room for new computations but also to support the distinction of relevant new from older information.

The literature on forgetting in the context of adult hippocampal neurogenesis, is more complex than this one aspect, though, and it might well be that those findings point to the functions of newborn neurons, for which we still lack a comprehensive idea (Tran et al., 2019). The same applies also to the findings that the new cells are relevant for memory consolidation (Kitamura and Inokuchi, 2014), recall and reconsolidation (Lods et al., 2021) and post-learning modifications (Luchetti et al., 2021). Intuitively, these observations harmonize well with the described core functionality, on further inspection many questions arise. These relate especially to the question of the temporal dimensions of the contribution that the new neurons make to the network. Are these only part of the initial computation at the “gateway to memory,” in the sense of new cells acting as “gatekeepers” at this gateway, or is that initial function intricately linked to consecutive and more lasting consequences, extending beyond that critical period? The latter seems likely but is much harder to address and has as yet been hardly studied.

## Turnover Versus Longterm Effects of Adult Neurogenesis

The new neurons in the hippocampus do not contribute to an immediate turnover of cells. Over long periods, a slow turnover might in fact occur (Spalding et al., 2013). Modeling has revealed that this is actually beneficial for the computation involving new neurons (Appleby et al., 2011). This however implies that the new cells are part of the network for much longer periods of time than their initial contribution during a phase of increased synaptic plasticity requires. After this period they blend into the existing network, but thereby also lastingly change it. This prolonged network effect is only poorly understood. It seems plausible that these effects are distinct from the original effects and they might relate to the observations that relate to recall and reconsolidation, but no details are known.

## Age-Related Changes in Cell Numbers and Developmental Dynamics

With aging, adult neurogenesis declines (Kuhn et al., 1996; Ben Abdallah et al., 2010; Encinas et al., 2011) and in human samples after puberty only small number of radial glia-like cells or cells with canonical neural stem cell markers are found (Terreros-Roncal et al., 2021). Inter-individual variation is likely to be large. But while the population of stem cells might ultimately disappear, intermediate progenitor cells (at least based on the expression of proxy markers like Doublecortin) have been detectable even in the oldest specimens, up to 100 years of age (Knoth et al., 2010).

If adult neurogenesis would go to zero altogether this would raise the question, like in the case of human adult neurogenesis, how the computational task that is deemed essential and relies on new neurons, continues to be achieved, once no new cells are available any longer. This has stimulated the hypothesis that in old age of rodents (and at relatively much younger age in humans) neurogenesis in the hippocampus becomes decoupled from stem cells and their proliferation but draws from a reservoir of later-stage cells that were produced earlier in development and rest until needed (Kempermann et al., 2018). This concept of immature neurons or “neurons in waiting” extends far beyond the hippocampus and has been suggested for the cortex (Bonfanti and Nacher, 2012; La Rosa et al., 2020). In theory this harbors the possibility that there is a functional contribution of newborn neurons also in regions previously considered non-neurogenic because of their lack of stem cells. For the hippocampus this hypothesis has not yet been tested experimentally, but the point is that scenarios are possible in which adult neurogenesis assumes the proposed functional role, despite an appearance that differs from the one familiar from mice.

If correct, this idea means that the relationship between function and regulation might change over time. Recruitment of new cells occurs postmitotically (Tashiro et al., 2007), but overall behavioral activity (especially locomotion) can at least on the short term strongly stimulate precursor cell proliferation (van Praag et al., 1999). This latter effect is so strong that many experimental studies addressing network effects have boosted neurogenesis through wheel running in rodents in order to increase the measurable output. At the behavioral level, however, there were subtle differences in the functional consequences that could be attributed to the contribution of adult neurogenesis enhanced either through voluntary wheel running or environmental enrichment (Garthe et al., 2016).

## A Life-Course Perspective on Adult Neurogenesis and Its Functions

From an evolutionary perspective the early and acute link between physical activity and the regulation of neurogenesis might point to the fact that cognition is strongly dependent on experience of the world, which for animals (and our ancestors that did not yet have smartphones) has been invariably linked to movement within that world (Kempermann et al., 2010). Action in the sense of movement and beyond always precedes cognition. With increasing experience and past network adaptations this stimulus might become less necessary, because the foundations have been laid. Old animals might have “seen it all” and require less plasticity. At the same time, their continued activity might have maintained their potential neurogenesis at a higher level so that they, should unexpected challenges arise in oldest age, still have more room for network plasticity (Kempermann, 2008). This “neurogenic reserve hypothesis” relates considerations about the functional relevance of adult neurogenesis, which tend to focus on short periods of time, to a life-course perspective and acknowledges that the process might change considerably over time without, however, losing its relevance. The idea of the “neurons in waiting” is in line with this concept but both remain to be proven, especially in their conjunction.

## Adult Neurogenesis and Affective Behaviors

The hippocampus is not only involved in learning and memory but, as part of the limbic system, also in affective behaviors. Both aspects are not independent of each other (Anacker and Hen, 2017). Emotional context, for example, are critical for the evaluation of incoming information for processing and subsequent storage.

But in addition to the emotional input on cognition, adult neurogenesis also appears to be involved in decision making that anticipates future rewards (Seib et al., 2021). As reward signals are involved in memory consolidation and reactivation in the hippocampus (Singer and Frank, 2009), the finding that adult-born neurons contribute to learning in situations of uncertainty about future reward adds a novel perspective (Seib et al., 2020). This example underscores that the conceptually different functions that can be ascribed to adult neurogenesis are interdependent and that this also includes functions that are related to emotional behaviors.

The same is true for the finding that adult neurogenesis conveys stress resilience, which is also not straightforward to subsume under the cognitive functions (Anacker et al., 2018).

In the case of post-traumatic stress disorder, the hippocampus plays an important role in the persistence and the persistent influence of emotionally loaded memories. Among other things, psychotherapy aims at “unlearning” such associations and prevent an over-generalization of fear (Guo et al., 2018). In the (by and large for many reasons still problematic) animal models of PTSD and depression (the related condition in which the affective state cannot be linked to learnable life events), adult hippocampal neurogenesis has often, but not unambiguously been shown to contribute to the disease-like phenotype (Besnard and Sahay, 2016). Reduced neurogenesis would be associated with the impaired ability to forget and to update information in an affect-loaded context. In one study, however, adult-born neurons stabilized the effects of aversive stimuli to distract from learning a task (Schoenfeld et al., 2021), so that the picture is not black and white.

Affective behaviors are mostly related to the function of the ventral hippocampus in rodents (corresponding to the anterior hippocampus in humans), which shows subtle differences in adult neurogenesis compared to the dorsal part, most notably a slightly reduced responsiveness to behavioral stimuli that regulate neurogenesis. Accordingly, in suicidal patients with major depression, fewer cells with neurogenesis-markers were detectable the anterior hippocampus (Boldrini et al., 2019). Whereas the findings of a role of adult neurogenesis in the remodeling of stress responses are experimentally well substantiated (Anacker et al., 2018), so that the contribution to the stress-related pathological states is plausible (Anacker and Hen, 2017), it is not clear to date to what extent adult-born neurons might be relevant for the actual emotions themselves (possibly extending beyond anxiety, etc.).

## Adult Neurogenesis and the Emergence of Individuality

If adult hippocampal neurogenesis allows the adaptation of the network in the hippocampus to the cognitive (and affective) in response to the experiences made and, based on these experiences, the predicted challenges ahead, it must follow that adult hippocampal neurogenesis contributes to the individualization of the hippocampus and its neuronal network. As such adult neurogenesis is a potent local driver of the connectome of the brain and hence might indirectly contribute to what makes each person’s brain unique (see for example the discussion in Abbott et al. (2020)).

In the reductionist context of a mouse study it could be shown that such individualization emerges also when both the genetic background and the outer environment are kept constant (Freund et al., 2013; Zocher et al., 2019). The driver of the individualization must lie in the so-called “non-shared environment”, that is the part of the environmental factor that is dependent on the actions of the individual. In that study, 20% of the inter-individual variance in adult hippocampal neurogenesis could be explained by the individual trajectories of explorative, territorial behavior (“roaming entropy”).^1^ A key question for future research in this context is to which extent adult hippocampal neurogenesis is indeed causal for the observed individualization and how this contribution interacts with other causal factors.

Nevertheless, the findings implicate that a substantial part of the functional consequences of adult neurogenesis might indeed be dependent on individual behavior, thereby contributing to what we become and are, due to our own actions (Kempermann, 2019). Roaming entropy has also been studied in humans, where it was found that greater levels of roaming entropy were associated with more positive affect and enhanced hippocampal-striatal connectivity (Heller et al., 2020).

## Discussion and Outlook

Over the past twenty years, after the first paper directly targeting the functional consequences of adult hippocampal neurogenesis (Shors et al., 2001), the field has matured and a rich picture has begun to emerge (Christian et al., 2014, 2020; Anacker and Hen, 2017; Tuncdemir et al., 2019; Abrous et al., 2021). As outlined above, this picture has clear threads and themes, for example around pattern separation, catastrophic interference and flexibility, but also still has many blank spots on its map. The broad scope of studies to date have revealed that the contribution of adult neurogenesis to function is dependent not only on the tasks and the species involved, but also on time, both with respect to time of neuronal development and time within the task at hand (Kim et al., 2012; Masachs et al., 2021). As function refers to events from cellular to behavioral, the question of how the different layers and points of time are linked becomes central.

How, for example, does a stem cell “know” that the hippocampus is about to “learn” and, hence, is in need of new neurons? How is the overall number of new neurons controlled or are these stochastic effects? How is the phase of increased synaptic plasticity controlled and why does it end? Assessing function across scales in an integrative way remains the major challenge for our field. At the same time, the comparatively clearly laid-out network structure in the hippocampus and the precisely identifiable position of adult neurogenesis within that system, bears many advantages over other systems, e.g., in the cortex. The multi-scale nature of neurogenesis-dependent functions is part of the even greater question of how to appreciate best the complexity of the function that adult-born neurons contribute, not only to the local network, but also the hippocampus and the entire brain. Ultimately this calls for an understanding of the networks that not only link the hippocampal sub-regions with each other but also the hippocampus with the rest of the brain. Given the central role of the hippocampus in overall brain function, an involvement of adult neurogenesis in a great number of processes can be discussed. Not all these ascriptions will be useful and some will be indirect or far-fetched, but in total also weak and more distant links will be relevant for a comprehensive understanding. To gain such wider insight we must:

1. learn more about the network effects of adult hippocampal neurogenesis, locally and in the greater context, under baseline and gain- or loss-of-function conditions, which calls for improved multi-electrode electrophysiology *in vivo* and *ex vivo* and the use of optogenetics and chemogenetics,
2. develop and apply improved behavioral tasks with multi-dimensional readouts that allow to assess realistically complex and ethologically relevant behaviors and relate them to specific aspects of adult neurogenesis,
3. build better, broadly informed computational models that allow specific functional predictions for both physiologic and pathological conditions, as well as, for example, across species boundaries, especially toward humans, and
4. develop a unifying hypothesis about the role of continued neurogenesis in the context of the (human) mind and brain.

## Data Availability Statement

The original contributions presented in the study are included in the article/supplementary material, further inquiries can be directed to the corresponding author.

## Author Contributions

The author confirms being the sole contributor of this work and has approved it for publication.

## Conflict of Interest

The author declares that the research was conducted in the absence of any commercial or financial relationships that could be construed as a potential conflict of interest.

## Publisher’s Note

All claims expressed in this article are solely those of the authors and do not necessarily represent those of their affiliated organizations, or those of the publisher, the editors and the reviewers. Any product that may be evaluated in this article, or claim that may be made by its manufacturer, is not guaranteed or endorsed by the publisher.

## Footnotes

1
https://www.brandmaier.de/roamingentropy/
